# Antiseptic efficacy of two mouth rinses in the oral cavity to identify a suitable rinsing solution in radiation- or chemotherapy induced mucositis

**DOI:** 10.1186/s12903-023-02884-5

**Published:** 2023-03-26

**Authors:** Paula Zwicker, Marcus Freitag, Florian H. Heidel, Thomas Kocher, Axel Kramer

**Affiliations:** 1 Institute of Hygiene and Environmental Medicine, Ferdinand-Sauerbruch-Str, University Medicine Greifswald, D-17475 Greifswald, Germany; 2 Internal Medicine C, Ferdinand-Sauerbruch-Str, University Medicine Greifswald, D-17475 Greifswald, Germany; 3 Department of Restorative Dentistry, Periodontology, Endodontology, and Preventive and Pediatric Dentistry, University Medicine Greifswald, Fleischmannstr. 4, D-17475 Greifswald, Germany

**Keywords:** Sodium hypochlorite, Hypochlorous acid, Octenidine dihydrochlorid, Granudacyn®, Octenidol® md, Antiseptic mouth rinse, Mucositis

## Abstract

**Objectives:**

Oral mucositis caused by intensive cancer chemotherapy or radiotherapy frequently results in pronounced damage of the oral mucosa leading to painful oral hygiene. To support oral care, antimicrobial effective mouth rinses may be used. Thus, the efficacy of a hypochlorite-based mouth rinse (Granudacyn®), assumed to be highly biocompatible because of the compounds being part of the natural pathogen defense, as possible antiseptic agent in case of oral mucositis was compared to that of an octenidine based antiseptic mouth rinse (Octenidol® md).

**Materials and methods:**

The study was conducted as monocentric, controlled, randomized, blind cross over comparative study on 20 volunteers. As a proof of principle, we performed the study on orally healthy subjects and not cancer patients. The efficacy was determined as reduction of colony forming units (cfu) on buccal mucosa as well as in saliva. After mouth rinsing for 30 s, samples were taken after 1 min, 15 min, 30 and 60 min. The lg-reduction was calculated as difference between lg-values of cfu pre- and post-treatment.

**Results:**

Both antiseptic mouth rinses induced a significant reduction of cfu on buccal mucosa and in saliva 1 min after mouth rinsing. The effect persisted up to 60 min. The octenidine based rinse was significantly superior to the hypochlorite-based rinse up to the last sample 60 min after rinsing. However, the known cytotoxicity of octenidine argues against its application.

**Conclusion:**

Within the limits of this study, due to its antiseptic efficacy, the hypochlorite-based rinse Granudacyn® can be regarded appropriate to support the oral hygiene in patients with a sensitive oral mucosa during an aggressive cancer chemotherapy and radiation treatment in case of oral mucositis.

## Introduction

Oral mucositis (OM), caused in cancer patients by irradiation or chemotherapy may be the most frequent non-hematological side effect of cancer treatment and a therapeutic challenge. At present, the treatment of oral mucositis is focused on pain management in combination with oral hygiene to reduce secondary infections. National guidelines for supportive treatment recommend standardized oral care by mouth rinsing with water or 0.9% saline solution, tooth cleaning with a soft toothbrush, cleaning of the interdental spaces with dental floss and/or interdental brushes and avoidance of noxious substances, accompanied by frequent clinical monitoring by the dentist [1]. Sharp edges should be removed from removable protheses or teeth, hopeless teeth be extracted, caries be excavated, broken fillings be replaced and prophylaxis performed to reduce gingival inflammation prior to intensive treatment options such as stem cell transplantation [2].

In patients undergoing chemotherapy, insertion of a removable dental prosthesis should be avoided during the treatment period [3, 4]. If a prothesis is already present, it should fit well to prevent ulceration of the oral mucosa. An inserted prosthesis has to be cleaned [5] outside the oral cavity concomitantly to oral care and it has to be removed while sleeping.

Although no guideline is available regarding the use of saline or sodium bicarbonate rinses in the prevention or supportive treatment of OM because of limited data, inert, bland rinses to increase oral clearance may be helpful for maintaining oral hygiene and improving patient comfort [6]. To prevent further inflammation due to the colonization of ulcerated mucosa by bacteria, fungi, and viruses, agents with anti-inflammatory and antiseptic efficacy appear promising [7]. Studies support the use of so-called multi-agent combination oral care protocols [3]. Agents with anti-inflammatory and mild antiseptic activity promote wound healing and reduce the incidence of chemotherapy- and radiotherapy-induced oral mucositis [8]. The benefit for supportive treatment of OM is e.g. proven for natural products [9] such as honey [10, 11], curcumin [12], aloe vera [13], propolis [14, 15] and chamomile [16]. In contrast, the use of highly effective but cytotoxic antiseptics such as chlorhexidine digluconate (CHG), which is considered the gold standard for mouth antisepsis, proved to be contraindicated. Despite its high antiseptic efficacy, CHG- based solutions did not significantly reduce the incidence and severity of mucositis compared to placebo [17]. Contrarily, CHG significantly enhanced mucositis and was reported as uncomfortable or painful [18] due to its cytotoxicity, which is 57.2-fold higher than that of povidone iodine [19]. At present, the use of antiseptic mouth rinses is exclusively recommended when mechanical oral hygiene is not feasible or during Candida infection or bacterial superinfection of mucosal lesions [6]. Therefore, an antiseptic rinse with improved tolerability regarding mucosal integrity, is clearly required.

While a broad variety of antiseptic oral rinses is available, well-tolerated antiseptics with sufficient efficacy are not specified. Due to the good tolerability of polihexanide [20], a mouth rinse based on polihexanide was proofed on volunteers as possible alternative; however, the antiseptic efficacy was too low [21]. Another promising alternative is sodium hypochlorite in combination with hypochlorous acid due to its antiseptic efficacy associated with anti-inflammatory activity [22]. Hypochlorite promotes healing by regulating cytokines and growth factors, killing pathogens, and modulating inflammation by different mode of actions [23, 24]. Its antiseptically effective concentration is significant less irritative than that of CHG and 0.5% hydrogen peroide [25]. Furthermore, no evidence for cytotoxicity could be found in a 3D model of the skin [26]. NaOCl/HOCl is highly effective against vegetative bacteria, bacterial spores, aspergilli, and enveloped viruses [27, 28] and was slightly more effective against biofilms than the combination of polihexanide with betaine [26]. Octenidine (OCT), introduced few years ago for oral cavity antisepsis, surpasses both CHG and polihexanide in microbicidal efficacy in vitro [29], but is more cytotoxic and irritant than CHG [19, 25].

Since no data on antiseptic efficacy in the oral cavity are available for NaOCl/HOCl, its commercial formulation Granudacyn® should be tested for its ability to reduce viable aerobic bacteria in comparison with a highly effective mouth rinse in vivo in healthy volunteers. This would prove its efficacy and suitability to ensure that use in cancer patients to support their limited oral hygiene is conceivable. We did not perform this study in cancer patients, because we lacked knowledge about the efficacy of Granudacyn®, but we assume that antibacterial efficacy of the mouth rinses may be comparable.

If the antiseptic efficacy of NaOCl/HOCl is clearly higher than for the polihexanide-based mouth rinse and ranges in the effectiveness between polihexanide and OCT based mouth rinses, hypochlorite can be considered for use during mucositis due to the lack of irritant potency.

## Materials and methods

### Trial design

The study was conducted as monocentric, controlled, randomized, blind cross-over comparative study on 20 volunteers (Table 1). The efficacy of the test product Granudacyn® mouth rinse was compared to Octenidol® md. Ringer solution served as negative control.

The reduction of bacteria was evaluated on the buccal cheek mucosa and in saliva of the volunteers. The test products were coded using the letters A (Granudacyn®), B (Octenidol® md) and C (Ringer solution).

Allocation ratio was 1:1. Every volunteer received every rinsing solution once in consecutive weeks.

**Table 1:** Use of test products in the cross over design

Volunteer	1st Week	2nd Week	3rd Week
1–10, group 1	A	B	C
11–20, group 2	B	C	A

### Participants

#### Inclusion criteria

20 healthy volunteers (Caucasian, male/ female) aged 21 to 51 years (mean 27.3 ± 6.3) were selected. Their participation was voluntary confirmed by a written consent. Preconditions were at least 25 teeth free of caries as well as a gingiva free of pocketing (periodontal screening index ≤ 1). The volunteers had to be able and willing to meet the requirements of the test plan. Volunteers were recruited from community.

#### Exclusion criteria

Volunteers with dental prosthesis as well as those with injuries of the mucosa of mouth and pharynx and/or severe impairment of the mouth, nose and throat area were excluded. Volunteers who had undergone a dental treatment within 2 weeks before the study or who had taken part in a clinical study within 30 days were not involved either. The same applies to pregnant woman, nursing mothers, alcoholics and drug addicts. The medication with mouth rinses and use of antiseptic mouth rinses had to be stopped at the latest 3 days before the study, and food (solid and liquid) was not allowed 2 h before the application of the test preparations. Furthermore, volunteers should not display intolerance to ingredients of the test preparations. For prevention of data corruption, systemic treatment with antibiotics, antifungals, antiviral and/or immune suppressive medication within 4 weeks before the study was an exclusion criterion. Since the main outcome of the study was bacteria reduction independently of species or genera, no oral swabs were taken to prove oral candidiasis.

Data were collected at Department of Restorative Dentistry, Periodontology, Endodontology, Preventive and Pediatric Dentistry, Dental School of the University Medicine Greifswald (Greifswald, Germany), from December 2018 to December 2019.

**Randomizing and decoding**: The test subjects received their consecutive numbers in order of their inclusion in the observational study by the principle investigator and kept them for the entire duration of the study. The test sheets and the test containers with the rinsing solutions or swabs were numbered accordingly and could thus be clearly assigned to the test persons. Furthermore, the rinsing solutions were blinded as given in Table 1 to enable blindness of the volunteers. Allocation of the mouth rinses and their sequence of use (Table 1) was performed a priori by another person. The volunteers were randomized in a 1:1 ratio using a simple randomization: The first ten volunteers were allocated to group 1, volunteers 11–20 were allocated to group 2 by the principle investigator. The volunteers were not informed about their allocation. The patients were blinded to the kind of mouth rinse they received, while the principle investigator was not blinded. For this reason, all results (count of colony forming units) were examined by a second person who was blinded to the type of intervention used.

#### Ethics approval and consent to participate

This study was approved by the Ethics Committee of the University of Greifswald (27.11.2018, Reg. No. BB 190/18). The participation was voluntary and anonymous and confirmed by an informed consent. All methods were carried out in full agreement with the World Medical Association Declaration of Helsinki.

### Test products

Mouth antisepsis was performed using the following commercially available products:

- Granudacyn® mouthwash (SastoMed GmbH, Innovate Therapy, Mölnlycke Health Care), ingredients: water, sodium chloride, 50 ppm hypochlorous acid, 50 ppm sodium hypochlorite.
- Octenidol® md (Schülke & Mayr GmbH), ingredients: purified water, macrogol glycerol hydroxystearate, glycerol 85%, cool mint aroma, sodium gluconate, sucralose, octenidine-HCl, citric acid, butylhydroxytoluol.
- Ringer solution as negative control.

### Interventions

At the beginning of the study baseline status of each volunteer was documented. The complete status of teeth and personal history regarding concomitant diseases, medication, habits, incompatibilities/ allergies as well as age and gender were recorded.

The study was subdivided into 3 study periods, in which each one of the two mouthwash solutions as well as Ringer solution had to be used.

The volunteers received the preparations to be tested on 3 dates each (cross-over, consecutive weeks) for a single application.

To exclude overlay effects, an interval of at least 7 days between each of the 3 dates was included.

After collection of the baseline sample, a 30-second mouthwash was carried out. The following samples were taken 1 min, 15 min, 30 min and 60 min after the end of rinsing.

### Outcomes

As the primary outcome, the lg- reduction of the total bacteria count was calculated as differences between the lg pre-values and lg post-values. Secondary outcomes were the differences between the rinses at similar time points and the acceptance of the test solutions.

### Microbiological techniques

**Fig. 1:**
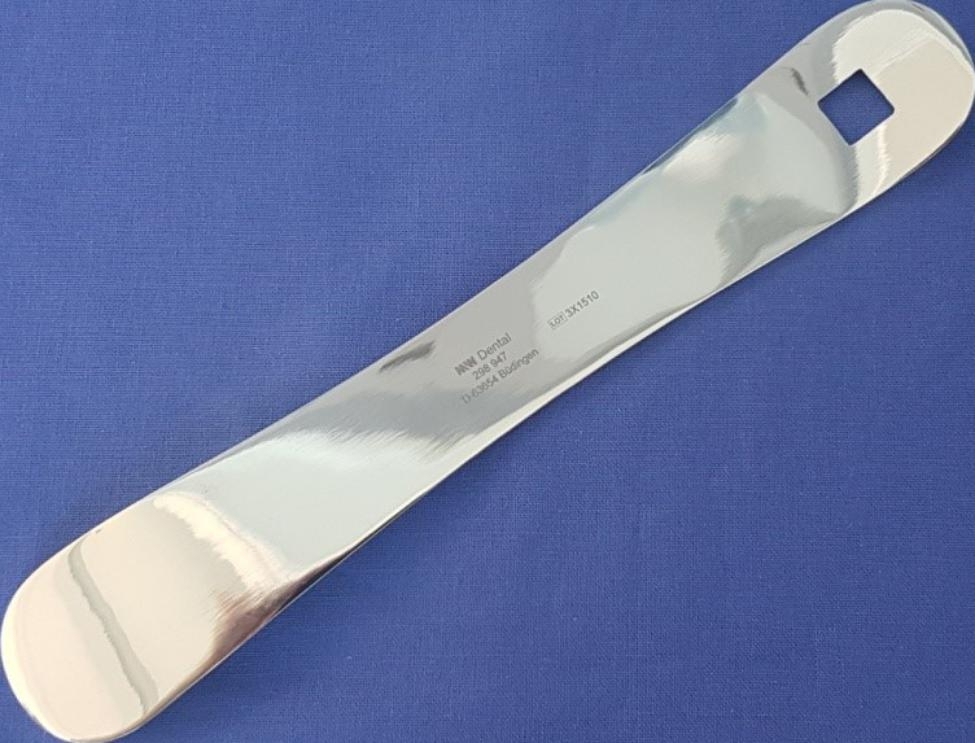
Sterilizable stainless steel template for standardized sampling within an area of 1 cm^2^

The number of colony forming units (cfu) was determined by swabbing the cheek mucosa on the right and on the left side at tooth 36 level and tooth 46 level. A slight pressure was applied to the mucous membrane and the area of sampling was limited to 1 cm^2^ using a stencil (Fig. 1). Furthermore, a saliva sample was taken using 20 ml Aqua as rinsing liquid. The rinsing was carried out for 30 s and was spat out into a sterile beaker afterwards. Samples were taken before and 1 min, 15 min, 30 min and 60 min after mouth rinsing.

After sampling, the swab was flushed in 0.9% NaCl and the supernatant plated on Columbia agar plates (BD Becton Dickinson Corporation, Franklin Lakes, NJ, USA). One part of the saliva sample was mixed with nine parts on a previously validated inactivator containing 3% Tween 80, 0.3% lecithin, 0.1% histidine and 0.1% sodium thiosulfate. The respective dilutions were plated onto Columbia blood agar plates following incubation for 48 h at 37 °C in a microaerophilic atmosphere. Afterwards, aerobic cultivated cfu (total bacteria count without distinction of colony morphology, species, etc.) were counted.

### Assessment of acceptance

In parallel, as secondary outcome, the acceptance of the test solutions was evaluated using a questionnaire. Volunteers were asked for acceptance in general (pleasant, tolerable, intolerable, disgusting), taste quality (sweet, sour, salty, bitter, sharp, refreshing, tasteless), influence on taste (reduced, unchanged) and saliva secretion (unchanged, increased, reduced).

### Sample size and statistical analysis

Sample size was calculated using G*Power 3.1 (University Düsseldorf). Since the study is an explorative study, the total sample size was calculated with an effect size of 0.4, an α error of 0.1 and a power of 0.9. for a comparison of the cfu before and after rinsing (5 groups). A total sample size of 85 was calculated to give an actual power of 0.9.

For comparison of the bacterial load before and after rinsing with Ocentidol® md, Granudacyn® or the placebo (Ringer solution), statistical evaluation was carried out using GraphPad Prism 4.00 using a one-way ANOVA with Dunn’s multiple comparison test. For comparison of effects between the different rinses at similar time points, one-way ANOVA with Tukey’s multiple comparison test was used.

## Results

### Participant flow and baseline data

In complete, 20 volunteers were enrolled to the study, ten at a time were assigned to each of the two groups enabling a cross-over design of the study. No volunteer was excluded. All of them completed the study.

Each group tested all of the three rinses. Volunteers were recruited from March 2019 to June 2019. The follow up started on July 2019 and ended in December 2019 after all volunteers received all rinses.

Mean age of the participants was 27.3 ± 6.3 years (group 1: 25.5 ± 3.3 years; group 2: 29.1 ± 8.0 years). In group 1, 40% of the volunteers were male whereas in group 2, 0% were male.

### Antiseptic efficacy

The colony morphology was very diverse for all samples. Colonies exhibiting a morphology specific for *Candida albicans* (filamentous colonies [30]) were not observed.

For analysis of the antiseptic efficacy, data from both groups were combined for each rinse (n = 20 for every rinse and time point).

Granudacyn® resulted in a lg- reduction of 0.94 ± 0.42 and 0.94 ± 0.56 (cheek right/ left) and 0.89 ± 0.45 (saliva) 1 min after rinsing. This effect was slightly increasing up to 30 min (Table 2) and then decreasing to 0.76 ± 0.66 and 0.65 ± 0.63. The reduction of cfu was significant compared to control.

The lg-reduction for Octenidol® md was about 2.29 ± 0.91 (cheek right), 2.26 ± 0.66 (cheek left) and 2.11 ± 0.66 in the saliva. The effect was consistent (cheek) up to 15 min and afterwards slightly decreasing (Table 2). In the saliva, decreasing effects were already observed at 15 min.

Both test products were significantly effective in reducing cfu for up to 60 min after rinsing.

**Table 2:** Mean lg-reduction for 20 test persons using Granudacyn® mouthwash, Octenidol® md mouthwash and Ringer- solution in comparison to the control. *p < 0.05, **p < 0.01, ***p < 0.001

		Lg-reduction			
Test Product		1 min	15 min	30 min	60 min
Granudacyn® mouthwash	Cheek right	0.94 ± 0.56	0.97 ± 0.556	1.00 ± 0.58	0.76 ± 0.66
	Cheek left	0.94 ± 0.42*	1.02 ± 0.52**	0.89 ± 0.55*	0.65 ± 0.63
	Saliva	0.89 ± 0.45**	0.91 ± 0.40**	0.85 ± 0.42*	0.70 ± 0.41*
Octenidol® md mouthwash	Cheek right	2.29 ± 0.91***	2.28 ± 0.91***	2.13 ± 0.90***	2.12 ± 1.00***
	Cheek left	2.26 ± 0.66***	2.32 ± 0.90***	2.25 ± 0.85***	2.14 ± 0.87***
	Saliva	2.11 ± 0.66***	1.85 ± 0.70***	1.79 ± 0.68***	1.64 ± 0.73***
Ringer solution	Cheek right	0.26 ± 0.42	0.16 ± 0.39	0.13 ± 0.43	0.09 ± 0.40
	Cheek left	0.21 ± 0.36	0.25 ± 0.44	0.14 ± 0.37	0.02 ± 0.25
	Saliva	0.19 ± 0.26	0.09 ± 0.27	0.17 ± 0.35	0.05 ± 0.26

There was no statistically significant difference in cfu (cheeks) between the negative control (ringer solution) and Octenidol® md (p = 0.12) and between Octenidol® md and Granudacyn® (p = 0.20) before rinsing (F = 6.817, p = 0.0016, r^2^ = 0.1044). There was a statistically significant difference between the negative control and Granudacyn® (p < 0.001).

One minute after rinsing until 60 min after rinsing, there were statistically significant differences between the negative control and Granudacyn (p < 0.0001) or Octenidol® md (p < 0.0001) as well as between Granudacyn® and Octenidol® md (p < 0.0001).

For saliva samples, no statistically significant results were obtained for the baseline between the negative control, Granudacyn® and Octenidol® md (F = 1.231, p = 0.2997, r^2^ = 0.04). One minute after rinsing up to 60 min after rinsing, there were statistically significant differences between the negative control and Granudacyn® or Octenidol® md as well as between Granudacyn® and Octenidol® md (p < 0.01 to p < 0.0001).

### Acceptance

Granudacyn® was judged tolerable by 15 test subjects. It was perceived as disgusting by 4 persons, as pleasant by one person. Additionally, 20 out of 20 test subjects mentioned the dominant chlorous taste.

Octenidol® md received the following evaluation: 12x tolerable, 8x pleasant.

The taste of Granudacyn® was evaluated rather differently: 7x salty, 5x bitter, 6x neutral, 1x pungent and 1x sweet. In contrast to Granudacyn®, Octenidol® md was judged as refreshing by 11 test subjects and as pungent by 8 persons. Just 1 volunteer perceived it as sweet.

## Discussion

Because of their highly antimicrobial efficacy, CHG and OCT are often used in antiseptic mouth rinses. However, these conventionally used rinses showed cytotoxic effects impeding their use in the context of oral mucositis. An alternative mouth rinse containing Citroxx revealed only low antimicrobial effects with a reduction of 0.22–1.36 lg levels [31]. A mouth rinse with a high cytocompatibility and an acceptable antimicrobial efficacy thus would be a real alternative for an accompanying treatment of OM and possibly also for its prevention.

In the presented study, we tested Granudacyn® with 50 ppm hypochlorous acid and 50 ppm sodium hypochlorite for a potentially use in patients suffering oral mucositis. Intentionally, only orally healthy volunteers were allowed to take part in the trial to first verify the efficacy and compatibility in a pre-test to ensure a potential use in cancer patients.

The use of hypochlorous acid mimics the naturally occurring antibacterial defense of macrophages and is therefore considered to be non-cytotoxic [32, 33]. Additionally, hypochlorite is anti-inflammatory active [22–24] and in combination with its antiseptic efficacy it is established for antisepsis of chronic wounds [20].

The study revealed a significant reduction of microorganisms (total aerobic bacteria count) in the oral cavity and importantly an effectiveness lasting up to 60 min with only a slight decrease. The prolonged effect of hypochlorite confirms results of Gottardi and Nagl [34]. However, Octenidol® md mouth rinse was significantly more effective at all tested time points. But, even with higher antibacterial efficacy, Octenidol® md is not a suitable mouth rinse with regard to severe oral mucositis because of its cytotoxicity [19, 25]. Moreover, its contracting effect on the arterioles can negatively impact the blood circulation and therefore results in adverse effects on oral wound healing. This effect is less pronounced when using irrigation solutions containing alcohol, however, the microcirculation of the tissue may also be influenced [35].

Since the study functions as a pre-test, only the total bacteria count was evaluated. However, a distinction of bacteria genera or even species would have been given more insight into the mode of action of both mouth rinses. Since hypochlorous acid is part of the natural defense of pathogens, a diverse reaction towards different bacteria genera may be possible. Since oral candidiasis seems to be a risk factor for early development of severe oral mucositis [36] knowledge about the antimicrobial efficacy of a mouth rinse against *Candida* spp. would give more detailed information for mucositis prevention.

A further limitation is the assessment of the antiseptic only on buccal mucosa and saliva but not at the dorsum of the tongue, which would have given further insights into the efficacy of the rinses especially on anaerobic microorganisms. But, even with its lower antimicrobial efficacy in comparison to Octenidol® md, Granudacyn® might be preferred since its higher biocompatibility on the already stressed oral mucosa [33]. Compared to a polihexanide based mouth rinse [21], Granudacyn® was more effective in the present study. As other mouth rinses, rinsing with Granudacyn® led to a long-lasting effect up to 60 min as shown. To prevent further inflammation due to bacterial regrowth, this depot-effect is a plus.

However, all conducted analyses were endpoint analyses after a single application. On the one hand, using healthy volunteers thus enables a cross-over study directly comparing the efficacy of the mouth rinses, on the other hand, mucositis in cancer patients may progress over time, thus a long-term application without cross-over design may provide a more realistic insight.

Furthermore, the slightly chlorous taste of Granudacyn® described by the volunteers repeatedly subsided very fast after mouth rinsing and therefore was classified as tolerable. Octenidol® md, in contrast, influenced the taste for several hours, caused by its characteristic long-lasting and bitter taste.

## Conclusion

The antiseptic efficacy of an OCT-based and a hypochlorite-based mouth rinse was compared for possible use during cancer chemotherapy and radiation treatment in case of severe mucositis.

Both solutions significantly decreased bacterial load in the oral cavity with Octenidol® md being more efficient than Granudacyn®. However, the efficacy of Granudacyn® ranged from the OCT-based to polyhexanide-based mouth rinses. Moreover, due to its higher biocompatibility it seems to be a promising alternative especially during phases of severe oral mucositis. However, studies for biocompatibility in vitro and in vivo including patients suffering from severe mucositis in order to determine a clinical benefit upon long term treatment are necessary.

## Data Availability

Original (de-identified) data are available from the corresponding author upon reasonable request.
